# Phenolic constituents in pepper (*Piper nigrum* L.) berries: UPLC-MS/MS analysis, antioxidant properties, antibacterial activity against *Pseudomonas fragi* and association analyzed by WCGNA

**DOI:** 10.1016/j.fochx.2025.102810

**Published:** 2025-07-18

**Authors:** Yiming Fang, Guiping Wu, Yunshuang Ding, Zhiqiang Niu, Jiale Feng, Rui Guo, Shuzhen He, Xu Wang, Hongying Zhu, Wenjiang Dong, Weicheng Hu, Xiaojian Jiang, Fenglin Gu

**Affiliations:** a Spice and Beverage Research Institute, Tropical Crops Genetic Resources Institute, Key Laboratory of Processing Suitability and Quality Control of the Special Tropical Crops of Hainan Province, National Center of Important Tropical Crops Engineering and Technology Research, Chinese Academy of Tropical Agricultural Sciences, Wanning 571533, China; b National Key Laboratory for Tropical Crop Breeding, Key Laboratory of Crop Gene Resources and Germplasm Enhancement in Southern China, Ministry of Agriculture and Rual Affairs, Key Laboratory of Tropical Crops Germplasm Resources Genetic, Improvement and Innovation of Hainan Province, Haikou 571101, China; c Sanya Research Institute of Chinese Academy of Tropical Agricultural Sciences, Haikou 571101, China; d Institute of Translational Medicine, School of Medicine, Yangzhou University, Yangzhou 225009, China; e School of Life Sciences, Huaiyin Normal University, Huaian 223300, China

**Keywords:** *Piper nigrum* L., Maturity stage, Phenolics, Antioxidant activity, Antibacterial activity, WGCNA, Multiple factor analysis

## Abstract

This study systematically investigated the maturation-dependent variations in phenolic composition and bioactivity of pepper (*Piper nigrum* L.) berries harvested at 210, 240, and 270 days after anthesis. Middle-maturity berries exhibited optimal accumulation of bioactive constituents, with total phenolic and flavonoid contents reaching 10.63 mg GAE/g DW and 0.16 mg QE/g DW, respectively. Free phenolic fractions, predominantly composed of flavonoids, demonstrated superior ORAC and DPPH radical scavenging activity. Glycosylated phenolics showed remarkable lipid peroxidation inhibition, while insoluble-bound phenolics displayed the most potent antibacterial effects against *Pseudomonas fragi*, as evidenced by minimal inhibitory and bactericidal concentrations. Weighted gene co-expression network analysis and multiple factor analysis, revealed significant positive correlations between specific phenolic classes and antibacterial efficacy. Furthermore, antioxidant capacity parameters showed strong associations with tannins and related compounds. These findings provide valuable insights into the maturation-dependent phytochemical dynamics and establish a robust analytical framework for elucidating structure-activity relationships in plant-derived bioactive compounds.

## Highlights

• Fresh pepper berries (*Piper nigrum* L.) serve as a potent natural source of antioxidants and antimicrobial compounds.
• Middle-maturity berries exhibited optimal accumulation of bioactive constituents.
• Glycosylated phenolic fractions exhibited remarkable lipid peroxidation inhibitory capacity.
• Insoluble-bound phenolic fractions showed superior antibacterial activity against *Pseudomonas fragi*.
• Integrated WGCNA and MFA analyses established significant structure-activity relationships in phenolic extracts.

## 1 Introduction

Pepper (*Piper nigrum* L.) is a perennial woody aromatic plant primarily cultivated in China, Vietnam, Indonesia, India, and Brazil (Takooree et al., 2019). In China, it is widely grown in the provinces of Hainan, Yunnan, and Guangdong. The berries are processed into different products at various stages of maturity, with black, white, and green pepper being the main products. These forms require harvesting the pepper at specific stages of development (Gu et al., 2013). Pepper has been added to foods since ancient times, not only as flavoring agents, but also as folk medicine and food preservatives (Zhang et al., 2022). In addition to imparting characteristic flavors, pepper prolong the storage life of foods by preventing rancidity through their antioxidant activity or through bacteriostatic or bactericidal activity (Kwon et al., 2015). Numerous studies have shown that phenolic compounds in spices and herbs significantly contribute to their antioxidant and pharmaceutical properties (Cai et al., 2004; Shan et al., 2005; Wu et al., 2006), and these compounds may also play a major role in antimicrobial effects (Shan et al., 2005). Pepper contains numerous bioactive compounds, among which phenolics, an important class of secondary metabolites, are influenced by factors such as plant species and growth environment. The biological activities of phenolics, including antioxidant, anti-inflammatory, antibacterial, and antitumor properties, have attracted considerable research interest (Dai & Mumper, 2010; Feduraev et al., 2019; Hu et al., 2023; Hu et al., 2025). Phenolic compounds in plants primarily exist in four forms: free, esterified, glycosylated and insoluble-bound (Ross et al., 2009). Among these, the free form is relatively uncommon, as phenolics are usually bound to large molecular structures within the cell-such as cellulose, proteins, lignin, and polysaccharides-through ether, aldehyde, or ester bonds. However, most research to date has concentrated on the free form, with comparatively less attention given to the other three forms.

Natural phenolic compounds exhibit antimicrobial activity by disrupting cell membrane integrity, inhibiting the synthesis and metabolism of biomacromolecules, and through other mechanisms (Papuc et al., 2017). *Pseudomonas fragi*, a Gram-negative bacterium, is a major contributor to spoilage in frozen beef, pork, chicken, and the deterioration of milk under aerobic conditions. *P. fragi* can thrive and reproduce rapidly at low temperatures, breaking down proteins and fats through active catabolic metabolism, which ultimately leads to significant food waste (Li et al., 2022). Antibiotics, one of the most important discoveries of the 20th century, are widely used in clinical, agricultural, and livestock sectors. However, the widespread use of antibiotics and other antimicrobial agents, including food preservatives, has contributed to the emergence of multidrug-resistant bacteria (Baquero et al., 2014). Therefore, there is an urgent need to develop natural antimicrobial agents as alternatives to antibiotics. Pepper has been added to food since ancient times, not only as a flavoring agent but also as a folk medicine and food preservative (Nakatani, 2000; Zhang et al., 2024). In addition to imparting characteristic flavors, pepper extends the shelf life of food by preventing rancidity through its antioxidant properties, as well as its bacteriostatic or bactericidal activities (Shan et al., 2007). Pepper, the most widely consumed spice, is rich in essential nutrients and bioactive compounds. The development and ripening of its berries involve a series of complex physiological and structural changes. Betta et al. (2018) reported that the phenolic content and antioxidant capacity of most fruits are higher in the unripe stage than at full ripeness, however, the opposite trend was observed during the ripening of tomatoes and strawberries (Oszmiański et al., 2018). To the best of our knowledge, limited information exists regarding the changes in phenolic composition, antioxidant capacity, and antibacterial activity against *P. fragi* in pepper during ripening.

The objective of this study was to evaluate the effect of maturity on the content of four phenolic fractions and assess their antioxidant capacity using oxygen radical absorbance capacity (ORAC), 1,1-diphenyl-2-picryl-hydrazyl (DPPH) scavenging activity, lipid peroxidation inhibition, and antibacterial activity against *P. fragi*. In addition, potential correlations between antioxidant and antibacterial properties and phenolic content were explored using weighted gene co-expression network analysis (WGCNA). Key phenolic compounds were identified from highly correlated modules for further verification and interpretation. This research is the first to report on the bioactive functions of pepper at different maturity stages, with findings that may improve the efficient utilization of pepper and support the development of natural antioxidants and meat preservatives.

## 2 Materials and methods

### 2.1 Plant material

Fresh pepper berries (*P. nigrum* L.) were collected at 210, 240, and 270 days after anthesis in 2023 from the Spices and Beverages Research Institute, Chinese Academy of Tropical Agricultural Sciences (Wanning, Hainan, China), and categorized as middle-maturity (MM), late-maturity (LM), and full-maturity (FM), respectively. The berries were stored in plastic bags at −80 °C until further analysis.

### 2.2 Chemicals and reagents

DPPH and 6-hydroxy-2,5,7,8-tetramethylchroman-1-carboxylic acid (trolox) were purchased from Yuanye Bio-Technology (Shanghai, China). Sodium hydroxide (NaOH), hydrochloride, butyl hydroxy anisole (BHA) and butylated hydroxytoluene (BHT) were purchased from Sinopharm (Shanghai, China). Gallic acid, quercetin, fluorescein, 2,2′-azobis (2-methylpropanimidamide) dihydrochloride (AAPH) and Folin-Ciocalteu's phenol reagent were purchased from Sigma-Aldrich (Sigma-Aldrich, St. Louis, MO, USA). HPLC-grade methanol was supplied by Merck (Darmstadt, Germany), and HPLC-grade water was purified using a Milli-Q system (Millipore, Bedford, MA, USA). All reagents were of analytical grade.

### 2.3 Extraction of free, esterified, glycosylated and insoluble-bound phenolics

The free, esterified, glycosylated and insoluble-bound phenolic compounds were obtained according to the method described by Wang et al. (2016) with slight modifications. The sample of fresh pepper berries was lyophilized, milled, and sieved through a standard sieve of 100 mesh. Twenty grams of ground freeze-dried berries were soaked in petroleum ether for 24 h, and extracted with 200 mL of methanol/water (7:3, *v*/v) for 1 h by ultrasound in the dark, and this extraction was repeated twice. After centrifugation, the supernatants were collected, and methanol was evaporated under vacuum. The resulting aqueous phase (Water Phase A) was acidified to pH 2 with 6 M HCl and extracted five times with ethyl acetate to isolate free phenolic compounds. The pooled ethyl acetate fractions were concentrated by rotary evaporation, and the dried extract (containing the free phenolic fraction) was redissolved in anhydrous methanol, brought to a final volume of 25 mL, and stored at −20 °C until analysis. The residual aqueous phase (Water Phase B), containing bound phenolics, underwent alkaline hydrolysis for 4 h at room temperature under N₂. The hydrolysis mixture consisted of 4 M NaOH, 10 mM EDTA, and 1 % ascorbic acid to minimize oxidation.

After acidification, the phenolic compounds released from soluble esters were extracted using the same procedure as described above. The final extract was redissolved in 25 mL of methanol and stored at −20 °C until further analysis, and it was considered the esterified phenolic compounds (esterified fraction). The remaining aqueous phase was then hydrolyzed with 6 M HCl for 30 min at 85 °C under nitrogen. Phenolic compounds released from soluble glycosylated compounds were extracted according to the previously described procedure. This extract was adjusted to 25 mL and stored at −20 °C until further analysis, and it was considered the glycosylated phenolic compounds (glycosylated fraction). The residue from the 70 % methanol extract was hydrolyzed with 4 M NaOH (containing 10 mM EDTA and 1 % ascorbic acid) under the same conditions as used for the esterified compounds. After acidification to pH 2 with 6 M HCl, the phenolic compounds released from the methanol extract residue were extracted as described earlier. The extraction was repeated three times, and the final extract was evaporated to dryness. The combined extracts were dried by rotary evaporation, redissolved in 25 mL of anhydrous methanol, and stored at −20 °C as the insoluble-bound phenolic fraction.

### 2.4 Determination of total phenolic and total flavonoid content

The total phenolic content (TPC) was determined using the Folin-Ciocalteu method (Obreque-Slier et al., 2023). Results were quantified against a gallic acid standard curve and expressed as milligrams of gallic acid equivalents per gram of dry weight (mg GAE/g DW). For total flavonoid content (TFC) analysis, a colorimetric assay was performed according to established methods (Ho et al., 2022; Lin et al., 2014). Quantification was based on a quercetin standard curve, with results expressed as milligrams of quercetin equivalents per gram of dry weight (mg QE/g DW).

### 2.5 Antioxidant activity analysis

#### 2.5.1 Oxygen radical absorbance capacity assay (ORAC)

The ORAC assay was conducted following the method of Koffi et al. (2015) with a slight modification. All reagents were prepared in a PBS buffer solution (pH 7.4). In the reaction system, 50 μL of 0.01 mg/mL extract and 100 μL of 70 nM sodium fluorescein (FL) solution were added and incubated at 37 °C for 10 min. Subsequently, 50 μL of 0.1 M AAPH solution was introduced. Ascorbic acid, gallic acid, BHA, and BHT served as positive controls. The result is obtained by calculating the area under the fluorescence curve, and it is expressed as μmol Trolox equivalents (μmol TE L^−1^).

#### 2.5.2 DPPH· radical scavenging activity assay

The DPPH radical scavenging activity was determined following the method of Fernandez-Orozco et al. (2011) with slight modifications. Briefly, 40 μL of samples with different gallic acid equivalent concentrations were mixed with 190 μL (200 μM) of fresh DPPH solution. The mixture was vortexed thoroughly and incubated in the dark at room temperature for 30 min. Absorbance was then measured at 517 nm using a microplate reader.

#### 2.5.3 Lipid peroxidation inhibitory capacity assay

Lipid peroxidation inhibitory capacity was measured by the TBA method (Alvarenga et al., 2022) with a slight modification. The reaction mixture consisted of 0.2 mL yolk suspension, 0.2 mL samples, and 0.2 mL 25 mM FeSO_4_⋅7H_2_O and incubated at 37 °C in the dark for 30 min. To terminate the reaction, 0.2 mL of 15 %TCA and 0.4 mL of 0.8 % TBA were added, followed by heating at 95 °C for 15 min. The mixture was then centrifuged at 8000 rpm for 15 min, and the supernatant was measured at 532 nm.

### 2.6 UPLC-ESI-MS/MS analysis

The sample extracts were analyzed using a UPLC-ESI-MS/MS system (UPLC, ExionLC™AD, https://sciex.com.cn/) and a tandem mass spectrometry system (https://sciex.com.cn/). The UPLC column was an Agilent SB-C18 column (1.8 μm, 2.1 mm × 100 mm) while the mobile phase was comprised of solvent A (pure water with 0.1 % formic acid) and solvent B (acetonitrile with 0.1 % formic acid). The gradient program was set as follows: 95:5 *V*/V at 0 min, 5:95 V/V at 9.0 min, 95:5 V/V at 10 min, 5:95 V/V at 11.1 min, 5:95 V/V at 14.0 min. The flow rate was set to 0.35 mL/min and the column oven was maintained at 40 °C. The injection volume was 2.0 μL. The effluent was alternatively connected to an ESI-triple quadrupole-linear ion trap (QTRAP)-MS. The electrospray ionization (ESI) source parameters were set as follows: ion source temperature, 500 °C; ion spray voltage (IS), 5500 V (positive ion mode) and − 4500 V (negative ion mode); ion source gases I (GSI) and II (GSII), and curtain gas (CUR) were set at 50, 60, and 25 psi, respectively; and collision-activated dissociation (CAD) was set to high. QQQ scans were performed using multiple reaction monitoring (MRM) with nitrogen as the collision gas at medium pressure. Declustering potential (DP) and collision energy (CE) for each MRM transition were optimized individually. Specific MRM transitions were monitored for each period based on the metabolites eluted during that time. Then, based on the accurate mass of metabolites, MS^2^ fragment ions, isotopic distribution of MS^2^ fragments, and retention time (RT), an in-house developed intelligent MS^2^ spectral matching method by Metware Metabolomics was used to perform a comprehensive match between the MS^2^ spectra and RT of metabolites detected in the samples and those stored in the company's database. The Metware database was constructed using reference standards, public libraries, and literature reports. By analyzing the fragmentation patterns of various metabolites from these sources, the database has been manually curated and currently contains over 4400 compounds.

### 2.7 Antibacterial activity analysis

#### 2.7.1 Minimum inhibitory concentration (MIC) and minimum bactericidal concentration (MBC)

The MIC and MBC of the extracts were determined by agar dilution method (He et al., 2022). In brief, 2 mL of pepper extracts at different concentrations were mixed with 18 mL of nutrient agar medium to achieve final concentrations of 0.625, 1, 1.25, 2, 2.5, 4, 5, and 8 mg/mL. Sterile water and 1 % ethanol were used as blank and negative controls, respectively. After the medium solidified, 200 μL of bacterial suspension (approximately 1 × 10^6^ CFU/mL) was evenly spread on the cooled medium and incubated at 30 °C for 24 and 48 h. The MIC was defined as the lowest concentration that inhibited visible bacterial growth. The MBC was defined as the lowest concentration of extract at which *P. fragi* showed no growth.

#### 2.7.2 Particle size measurement

To investigate the particle size changes induced by the extracts, bacterial suspensions were measured using a Zetasizer Nano ZS90, following the method of (Kalily et al., 2017). Bacteria were grown in LB medium for 12 h at 30 °C under shaking conditions, and the cell suspensions were incubated with the MIC of different samples for 8 h. After centrifugation at 8000 rpm for 6 min at 4 °C, the bacterial cells were washed three times with PBS and resuspended in 5 mL of PBS.

#### 2.7.3 Nucleic acid and protein leakage

Nucleic acid and protein leakage were performed by the previous literature (Ma et al., 2016). Bacteria were cultured to the logarithmic phase, washed three times, and resuspended in PBS. Four phenolic extracts at the MIC, along with 1 % ethanol and sterile water, were added to the bacterial suspension. The suspensions were incubated for 0, 2, 4, and 8 h, after which the supernatants were collected. The supernatants were then used to measure nucleic acid and protein leakage by recording absorbance at 260 and 280 nm, respectively.

#### 2.7.4 Reactive oxygen species (ROS) levels

The fluorescent probe DCFH-DA was used to determine intracellular ROS levels, as described by Liao et al. (2019). Bacteria treated with different extracts at the MIC, blank (sterile water), and 1 % ethanol were collected at 0, 2, 4, and 8 h. The bacteria were then washed three times with PBS and incubated with DCFH-DA for 30 min in the dark. ROS levels were measured using a fluorescence spectrophotometer (Hitachi F-3000, Japan), with an excitation wavelength of 485 nm and an emission wavelength of 528 nm and expressed as relative fluorescence intensity. Express the value of sterile water (Blank) as 100, and represent other test data as a percentage relative to the Blank by multiplying their ratios by 100.

#### 2.7.5 Cell membrane permeability

Propidium iodide (PI) and Calcein-AM were used to evaluate cell membrane integrity, following the method of Ning et al. (2022). Briefly, cell suspensions (1 × 10^8^ CFU/mL) were treated with the MIC of four extracts, 1 % ethanol, and sterile water for 8 h, respectively. The cells were then washed with saline and resuspended in 0.85 % (*w*/*v*) saline. Subsequently, the cells were stained with 10 μM PI and 10 μM Calcein-AM in the dark for 20 min. After staining, the cells were washed with sterilized saline and observed under a BX51 fluorescence microscope (Olympus, Tokyo, Japan).

#### 2.7.6 Cell morphological change

The intracellular changes and cell membrane integrity of *P. fragi* were observed using a laser confocal microscope and transmission electron microscopy. Followed the treatment as described as above, the bacterial cells were washed three times with PBS and processed according to the kit instructions before being observed under the LCM. For TEM analysis, *P. fragi* in the exponential phase were treated with the phenolic extracts at the MIC, 1 % ethanol, and sterile water at 30 °C for 8 h. Bacterial culture medium was applied to a copper grid and air-dried before the samples were observed under the TEM.

### 2.8 Association of total phenolic content, total flavonoid content, antioxidant activity, antibacterial activity and phenolic compounds via WGCNA

Total phenolic content, total flavonoid content, antioxidant activity and antibacterial activity were analyzed to assess their correlations with phenolic compounds. A metabolite co-expression network was constructed using the WGCNA R package (v 1.47) (Langfelder & Horvath, 2008), with a correlation matrix soft-thresholding power β of 13. After calculating the topological overlap measure (TOM) from the adjacency matrix, the dissimilarity TOM was used to generate a dendrogram. Modules were identified using the DynamicTreeCut algorithm and assigned distinct colors. Highly correlated metabolites were grouped into a single module.

### 2.9 Verification of WGCNA result via multiple factor analysis (MFA)

MFA was utilized to explore and validate the relationships between total phenolic content, total flavonoid content, antioxidant activity, antibacterial activity, and phenolic compounds within key modules identified through WGCNA. The FactoMineR package (Lê et al., 2008) was employed to conduct the MFA, which efficiently analyzed datasets containing multiple variable groups. MFA provided valuable insights into the complex interactions, enhancing the understanding of the roles of phenolic and flavonoid compounds in antioxidant and antibacterial activities.

### 2.10 Statistical analysis

All analyses were repeated three times. Principal component analysis (PCA) was conducted to cluster samples based on the peak areas of all identified metabolites using R package models (http://www.r-project.org/). Analysis of variance (ANOVA) was performed using SPSS 26.0 (SPSS Inc., Chicago, IL, USA), and the results are expressed as mean ± standard deviation. Significance levels were assessed using Duncan's multiple range test at a 5 % probability level (*p* < 0.05).

## 3 Results and discussion

### 3.1 The total phenolic content (TPC) and total flavonoid content (TFC)

The TPC and TFC in the four phenolic fractions of pepper at three different maturity stages are shown in Table 1. The TPC decreased from 10.53 mg GAE/g DW at the middle-maturity (MM) stage to 8.39 mg GAE/g DW at the full-maturity (FM) stage. Previously reported trends in phenolic content for acerola, yellow guava, and jambolan showed a sharp decline with increasing maturity (Betta et al., 2018). In this study, we observed a significant reduction in total phenolic content between the MM and FM stages (*p* < 0.05). However, there was only a slight change in TPC between the late-maturity (LM) and FM stages, a trend also observed in prior research (Takahashi et al., 2018). Among the four phenolic fractions, the free fraction was the predominant component, followed by the esterified and insoluble-bound fraction. Interestingly, the insoluble-bound fraction was the most abundant at the MM stage, alongside the free fraction, suggesting that berries at the MM stage may be a valuable source of phenolics. The TFC exhibited a similar trend to the TPC, ranging from 0.12 mg QE/g DW to 0.16 mg QE/g DW. The TFC initially decreased and then increased with fruit maturity. Overall, both TPC and TFC displayed similar trends, with TFC showing an initial decrease followed by a slight increase as the berries matured.

**Table 1:** Total phenolic and flavonoid content of the four fractions from fresh pepper berries at three maturity stages.

Maturity stages	Total phenolic content (mg GAE/g DW)				Total flavonoid content (mg QE/g DW)			
	Free	Glycosylated	Esterified	Insoluble-bound	Free	Glycosylated	Esterified	Insoluble-bound
MM	4.17 ± 0.13^a^	1.75 ± 0.42^a^	1.70 ± 0.08^b^	2.90 ± 0.71^a^	0.12291 ± 0.03429^a^	0.01009 ± 0.00309^a^	0.01231 ± 0.00305^a^	0.01566 ± 0.00447^a^
LM	3.20 ± 0.83^a^	1.40 ± 0.20^a^	2.63 ± 0.65^a^	1.60 ± 0.35^b^	0.08033 ± 0.02873^a^	0.00689 ± 0.00140^a^	0.01926 ± 0.00502^a^	0.00996 ± 0.00390^ab^
FM	3.78 ± 0.24^a^	1.62 ± 0.01^a^	2.02 ± 0.07^b^	0.96 ± 0.47^b^	0.10748 ± 0.03798^a^	0.00836 ± 0.00205^a^	0.01665 ± 0.00549^a^	0.00532 ± 0.00257^b^

Different lower case letters correspond to significant differences in different maturity stages at *p* < 0.05.

### 3.2 UPLC-MS/MS analysis of phenolic compounds

**Fig. 1:**
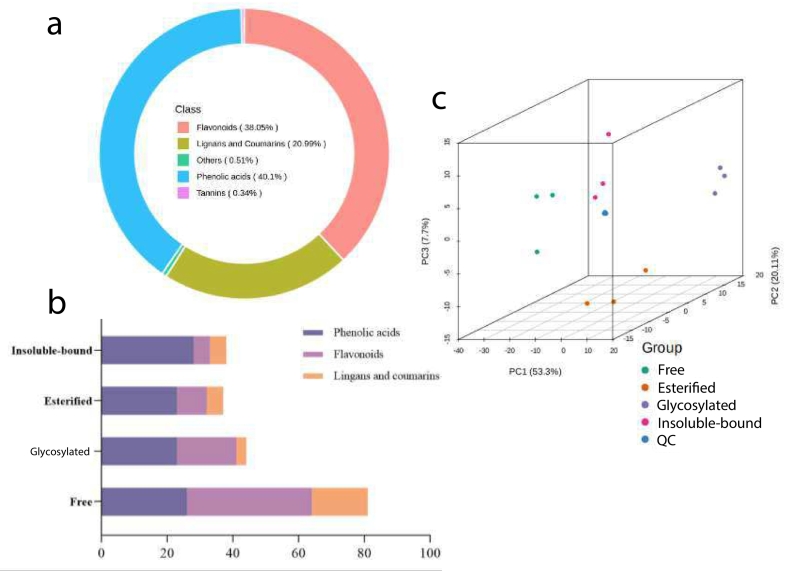
The profiles of the phenolics in MM pepper berries. (a) Classification of the 586 phenolic compounds in four extracts. (b) Classification chart of the high content phenolics in MM pepper berries. (c)3D PCA of phenolics profiles in four fractions.

The UPLC-MS/MS method was employed to identify four phenolic fraction of MM pepper extracts, resulting in the identification of a total of 586 compounds, including phenolic acids (40.1 %), flavonoids (38.05 %), lignans and coumarins (20.99 %), tannins (0.34 %), and others (0.51 %) (Table S1, Fig. 1). As detailed in Table S1, compounds exhibiting significant changes in relative abundance were selected for further analysis. Among the free phenolic compounds, apigenin-6-C- (2″-glucosyl) arabinoside, isoschaftoside, apigenin-6- C-(2″-glucuronyl) xyloside, kaempferol-3-O-glucorhamnoside were found to be relatively high. In the other samples, 2,4-dihydroxybenzoic acid, bis(2-ethylhexyl) phthalate, apigenin-8-C-Glucoside and 7-methoxycoumarin had relatively high concentration. Previous studies have classified pepper phenolics into enzyme-inactive and enzyme-active classes. Enzyme-inactive phenolic acids, such as vanillic acid, caffeic acid, butyric acid, protocatechuic acid, and salicylic acid (Variyar & Bandyopadhyay, 1994), were detected in this study. However, 3,4-dihydroxyphenylethanol glucoside and 3,4-dihydroxy-6-(N-ethylamino) benzamide, known substrates for polyphenol oxidase (PPO), were not found, possibly due to the extraction solvent used. Fig. 1b illustrated the distribution of different compound types in the four fractions. In three of the fractions (excluding the free fraction), phenolic acids (hydroxybenzoic and hydroxycinnamic acids) comprised more than 50 % of the identified substances, followed by flavonoids. In contrast, flavonoids constituted over 47 % of the total substances in the free fraction, with phenolic acids accounting for 32 %. Previous research suggested that phenolic acids in plants are predominantly present in bound forms, which contribute significantly to antioxidant activity, whereas free phenolics are primarily found as flavonoids, consistent with findings reported by Kang (2017).

### 3.3 Multivariate discrimination of phenolic compounds

To further explore the similarities and differences among the four phenolic fractions, principal component analysis (PCA) was performed. The PCA score plot revealed that principal component 1 (PC1) accounted for 53.3 % of the total variance, while principal component 2 (PC2) explained 20.11 %, and principal component 3 (PC3) contributed 7.7 % to the total variance (Fig. 1c). The PCA results clearly demonstrated a distinct separation between the free and glycosylated fraction. In contrast, the esterified and insoluble-bound fraction were highly overlapping, suggesting that these fractions are quite similar in terms of phenolic acid types and content.

### 3.4 Antioxidant activity

ROS, which can be harmful, are produced in the human body in the presence of oxygen. An imbalance between pro-oxidants and antioxidants leads to oxidative stress, a key factor in diseases such as diabetes, hypertension, and Parkinson's disease (Munteanu & Apetrei, 2021). Phenolic compounds, which are major secondary metabolites, are well-known for their potent antioxidant properties. All maturity stages and four phenolic fractions demonstrated antioxidant activity, as indicated by ORAC, DPPH radical scavenging assays and lipid peroxidation inhibition (Table 2). Previous studies have shown a strong correlation between antioxidant activity and phenolic content, a finding consistent with our results. The free fraction from berries at all ripening stages exhibited the highest ORAC and DPPH radical scavenging activities, ranging from 0.040 μM TE/L to 0.038 μM TE/L and from 0.63 mg/mL to 0.83 mg/mL, respectively. Although the free fraction had the highest content, their lipid peroxidation inhibition capacity was lowest at the MM and LM stages, with IC_50_ values of anti-lipid peroxidation capacity of 1.57 mg/mL and 1.56 mg/mL, respectively. The lipid peroxidation inhibition capacity of the free fraction improved at the FM stage, and the glycosylated fraction demonstrated the strongest inhibition at the MM and FM stages, with IC_50_ values of anti-lipid peroxidation capacity of 0.31 mg/mL and 0.32 mg/mL, respectively. All other fractions exhibited stronger lipid peroxidation inhibition than ascorbic acid except for the free fraction at the MM and LM stages. This variation may result from different substances exerting the main antioxidant effects and an increase in substances with a stronger inhibitory effect on lecithin oxidation during pepper ripening. The esterified fraction showed the highest phenolic content at the MM stage (1.75 mg GAE/g DW), but the strongest ORAC and DPPH radical scavenging activities were observed at the FM stage, with ORAC values similar to those of the free fraction. In this study, the TPC of insoluble-bound fraction decreased with maturity, and their lipid peroxidation inhibition and DPPH radical scavenging activity followed this trend. However, in the ORAC assay, the insoluble-bound fraction exhibited stronger activity at the MM stage compared to the LM and FM stages, though the difference was not significant. In summary, higher antioxidant activity was observed in the MM stage among the four phenolic fractions, likely related to increased flavonoid content at this stage. Further research is needed to identify the specific substances responsible for these differences.

**Table 2:** The antioxidant activities of the four fractions from fresh pepper berries at three maturity stages based on ORAC assay, IC_50_ of DPPH radical scavenging activity and IC_50_ values of anti-lipid peroxidation capacity.

Maturity stages	ORAC value (μmol TE/L)					IC_50_ of DPPH radical scavenging activity (mg/mL)					IC_50_ values of anti-lipid peroxidation capacity (mg/mL)			
	Free	Esterified	Glycosylated	Insoluble- bound		Free	Esterified	Glycosylated	Insoluble- bound		Free	Esterified	Glycosylated	Insoluble- bound
MM	0.040 ± 0.001^a^	0.029 ± 0.001^b^	0.024 ± 0.002^a^	0.032 ± 0.014^a^		0.63 ± 0.24^a^	3.5 ± 1.12^a^	1.29 ± 0.27^ab^	2.07 ± 0.35^a^		1.57 ± 0.54^a^	0.78 ± 0.27^a^	0.31 ± 0.28^a^	0.36 ± 0.02^b^
LM	0.036 ± 0.003^a^	0.032 ± 0.005^ab^	0.023 ± 0.001^a^	0.024 ± 0.001^b^		0.83 ± 0.27^a^	1.90 ± 1.09^ab^	0.90 ± 0.14^b^	2.22 ± 0.56^a^		1.56 ± 0.27^a^	0.41 ± 0.29^b^	0.54 ± 0.36^a^	0.40 ± 0.11^b^
FM	0.038 ± 0.001^a^	0.037 ± 0.005^a^	0.026 ± 0.002^a^	0.023 ± 0.003^b^		0.64 ± 0.03^a^	1.40 ± 0.11^b^	1.44 ± 0.20^a^	2.29 ± 0.3^a^		0.37 ± 0.15^b^	0.58 ± 0.26^ab^	0.32 ± 0.08^a^	0.76 ± 0.20^a^
	Positive control													
	ORAC value (μmol TE/L)					IC_50_ of DPPH radical scavenging activity (mg/mL)					IC_50_ values of anti-lipid peroxidation capacity (mg/mL)			
	Ascorbic acid	Gallic acid	BHT	BHA		Ascorbic acid	Gallic acid	BHT	BHA		Ascorbic acid	Gallic acid	BHT	BHA
	0.042 ± 0.005	0.001 ± 0.001	0.012 ± 0.001	0.099 ± 0.016		0.23 ± 0.01	0.14 ± 0.00	7.36 ± 0.32	0.28 ± 0.01		1.08 ± 0.05	0.67 ± 0.02	0.14 ± 0.02	0.016 ± 0.00

Different lower case letters correspond to significant differences in different maturity stages at *p* < 0.05.

### 3.5 Antibacterial activity against *P. fragi*

#### 3.5.1 MIC and MBC

The MIC and MBC of the four fractions of MM pepper phenolic extracts on *P. fragi* were presented in Table 3. The MIC and MBC values ranged from 1 to 4 mg/mL, indicating that all fractions exhibited antibacterial activity. The insoluble-bound fraction demonstrated the highest antibacterial efficacy, with both MIC and MBC values of 1 mg/mL. In contrast, the free fraction showed the lowest antibacterial activity, with MIC and MBC values of 2 mg/mL and 4 mg/mL, respectively. Previous studies have reported antimicrobial activities of water-ethanol extracts from *Myrtus communis* L. (Casaburi et al., 2015) and 3-carene (Tang et al., 2022) against *P. fragi*, with MIC values of 25 mg/mL and 8.7 mg/mL, respectively, indicating that pepper phenolic extracts possess stronger antibacterial properties than these other extracts.

**Table 3:** Antibacterial activity of the four fractions of MM pepper extracts against *P. fragi*.

Bacterial strain	Class name	MIC (mg/mL)	MBC (mg/mL)
*P. fragi*	Sterile water	+++	+++
	Ethanol (1 %)	+++	+++
	Free	2	4
	Glycosylated	1	2
	Esteried	1.25	1.25
	Insoluble-bound	1	1

Note:+++ indicates a large number of bacterial colonies.

#### 3.5.2 Particle size distribution

**Fig. 2:**
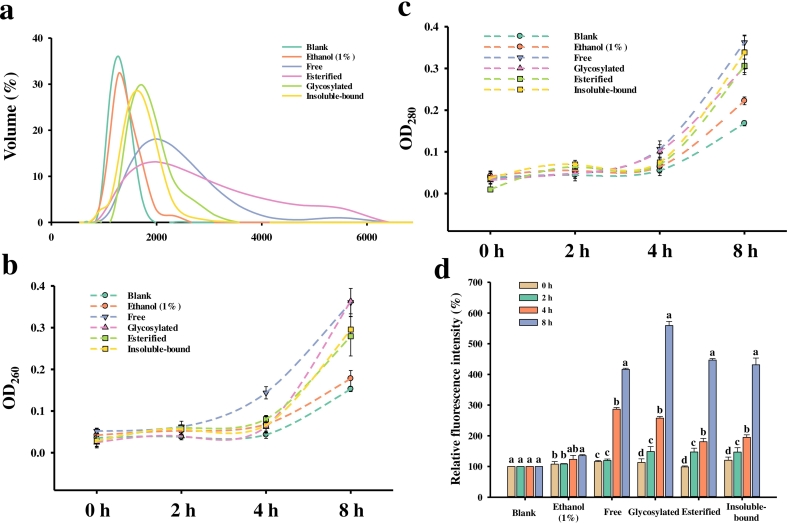
Comparison of the antibacterial activities against *P. fragi* of the MM pepper extracts. (a) Particle size distribution of *P. fragi* treated with four fractions for 8 h. (b) Nucleic acid leakage of *P. fragi* with four fractions. (c) Protein leakage of *P. fragi* with four fractions. (d) ROS levels. Bars indicate the standard deviation from triplicate determinations. Different letters in the same treatment indicate a statistical difference (*p* < 0.05).

The particle size distribution of treated *P. fragi* was presented in Fig. 2a. In the blank and ethanol groups, the bacterial suspensions primarily consisted of particles with a size around 1.5 μm. However, after treatment with the phenolic extracts, the particle size distribution shifted to the right, suggesting that the extracts may have induced bacterial aggregation. A similar phenomenon was observed in *Pseudomonas aeruginosa* treated with linalool (He et al., 2022; Liu et al., 2020). Notably, the size distribution peaks changed significantly with treatment by the free and esterified fraction. Furthermore, the free fraction resulted in a bimodal distribution with a large volume fraction, and bacterial aggregation was also confirmed by TEM.

#### 3.5.3 Leakage of nucleic acid and protein

Bacterial membranes are crucial for bacterial metabolism (O'Brien, 1967). Damage to the bacterial membrane leads to the leakage of small molecules first, followed by larger molecules such as DNA, RNA, and proteins (Shen et al., 2015). To evaluate changes in cell membrane permeability, nucleic acid and protein leakage were quantified by measuring absorbance at 260 nm and 280 nm, respectively (Fig. 2b, c). The results indicated a time-dependent increase in absorbance at both wavelengths, reflecting leakage. Notably, leakage of larger molecules (DNA, RNA, and proteins) from *P. fragi* treated with phenolic extracts began to accelerate within 4 h. This was followed by a rapid increase in absorbance between 4 and 8 h, suggesting substantial efflux of cellular components after 4 h. Similar observations were reported in previous studies involving cinnamon essential oil treatment of *Escherichia coli* and *Staphylococcus aureus* (Zhang et al., 2016). Campos et al. (2009) proposed that certain phenolic compounds, including p-coumaric, p-hydroxybenzoic, protocatechuic, caffeic, vanillic, and syringic acids, can permeate the cytoplasmic membrane, increasing permeability and promoting leakage of bacterial cellular components.

#### 3.5.4 ROS level in *P. fragi*

Excessive accumulation of ROS can oxidize essential cellular components, potentially leading to premature cell death (Wojtala et al., 2014). Previous research has shown that antibiotics can kill bacteria by increasing intracellular ROS through the Fenton reaction (Grant et al., 2012). In our study, we evaluated ROS accumulation in *P. fragi* following treatment with four phenolic extract fractions from MM pepper (Fig. 2d). All treatment groups exhibited time-dependent ROS accumulation, though with notable variability among fractions. ROS production followed a biphasic pattern: a gradual increase during the initial 2 h was followed by a dramatic surge between 4 and 8 h, with significant differences emerging among treatment groups (*p* < 0.05).

The glycosylated fraction induced the highest ROS production after 8 h of treatment—approximately five times higher than that of the control—followed by the esterified, insoluble-bound, and free fraction. Significant differences were found between the glycosylated, esterified, and free fraction (*p* < 0.05). This trend in ROS production was consistent with that of DNA and protein leakage, suggesting that the phenolic extracts exerted considerable bacteriostatic effects after 4 h. Similarly, Gao et al. (2023) reported a significant increase in ROS levels in *Hafnia alvei* following treatment with linalool, and similar results were observed in *E. coli* treated with silver nanoparticles (Xu et al., 2012).

#### 3.5.5 Laser confocal microscope

**Fig. 3:**
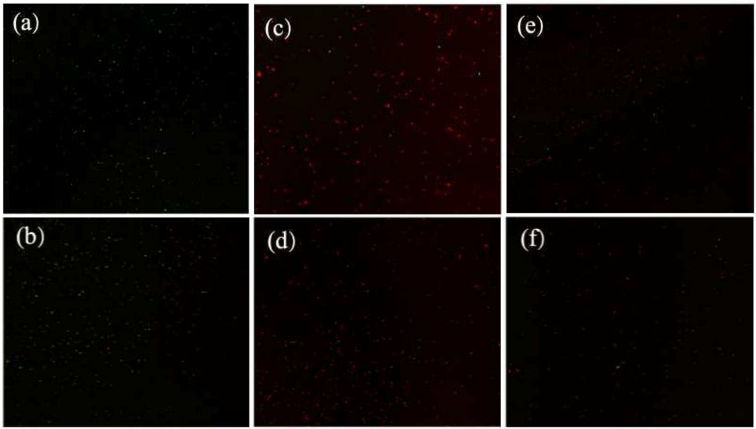
Images of *P. fragi* stained with Calcein-AM and PI, respectively, under a laser confocal microscope. (a) control group; (b) Treated with 1 % ethanol for 8 h. (c) treated with free fraction for 8 h; (d) Treated with glycosylated fraction for 8 h. (e) Treated with esterified fraction for 8 h. (f) Treated with insoluble-bound fraction for 8 h.

To further investigate the impact of different phenolic fractions on the cell membrane integrity of *P. fragi*, Calcein-AM and PI were used as fluorescent dyes. Calcein-AM, due to its hydrophobic nature, easily penetrates the cell membrane of living cells and is hydrolyzed by esterases into calcein, which emits strong green fluorescence and remains inside the cell. In contrast, PI is a red dye that cannot pass through an intact cell membrane; it only enters cells with damaged membranes, binding to DNA and emitting red fluorescence. The fluorescence color can range from red to orange to yellow, depending on the extent of membrane damage (He et al., 2023). The effects of the four fractions of MM pepper phenolic extracts on cell membrane integrity were illustrated in Fig. 3. The blank group (a) and the positive control group (b) exhibited significant green fluorescence and minimal red fluorescence, indicating that most cells maintained intact membranes. However, after 8 h of treatment with the phenolic fractions, there was a marked increase in red fluorescence, with limited green fluorescence remaining. Notably, the insoluble-bound fraction treatment group (f) showed a lower overall fluorescence density, suggesting fewer viable cells in this group. This result may be due to the strong efficacy of the insoluble-bound fraction in causing extensive membrane damage and cell fragmentation. Similar results have been reported with *Houttuynia cordata* crude extract on *Cronobacter sakazakii* (Chang et al., 2023) and carvacrol on *E. coli* and *Salmonella* (Ma et al., 2022).

#### 3.5.6 TEM analysis

**Fig. 4:**
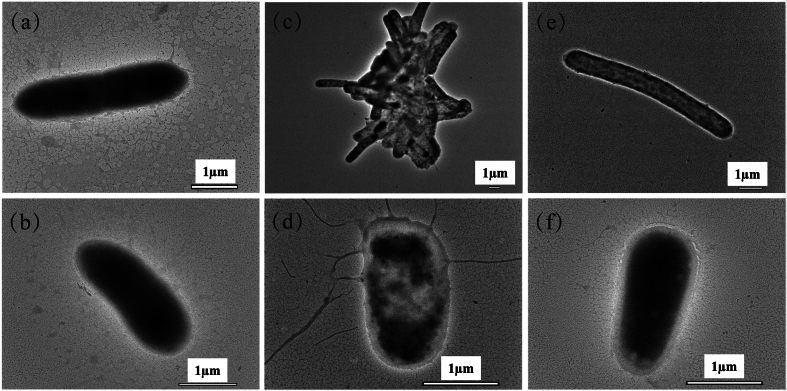
TEM images of *P. fragi.* (a) Control group. (b) Treated with 1 % ethanol for 8 h. (c) Treated with free fraction for 8 h. (d) Treated with glycosylated fraction for 8 h. (e) Treated with esterified fraction for 8 h. (f) Treated with insoluble-bound fraction for 8 h.

The TEM images in Fig. 4 illustrated the morphological changes in *P. fragi* cells treated with the four fractions of MM pepper phenolic extracts. In the control group and the 1 % ethanol treatment group, cells exhibited smooth surfaces, intact cytoplasm, and no observable damage (Fig. 4a-b). Upon treatment with the free phenolic extracts at MIC concentrations, cells displayed deformed rod-shaped structures, with noticeable release of internal components and aggregation into large clusters (Fig. 4c). Treatment with the glycosylated fraction at the same concentration caused blurring of cell boundaries and rupture of the cell membrane, leading to substantial leakage of intracellular contents (Fig. 4d). Cells treated with the esterified fraction exhibited significant elongation and leakage of cytoplasmic contents (Fig. 4e). Similarly, treatment with insoluble-bound fraction resulted in aggregation of cytoplasmic contents and loss of membrane integrity (Fig. 4f). These effects became more pronounced after 8 h of treatment, suggesting that the natural phenolic extracts exhibit a gradual bacteriostatic effect that ultimately leads to cell death. This occurs through disruption of membrane integrity and induction of ROS generation. The results indicated that pepper phenolic extracts reduced cellular activity or induce cell death by altering cell morphology, compromising membrane integrity, and causing the release of intracellular components to varying degrees.

Comparable effects were reported in previous studies involving the treatment of *P. fragi* with *Sedum aizoon* L. flavonoid extracts (Wang et al., 2022). Overall, the results confirm that pepper phenolic extracts have a significant antibacterial effect against the *P. fragi* strain (Wang et al., 2022). Overall, the results confirm that pepper phenolic extracts have a significant antibacterial effect against the *P. fragi* strain.

### 3.6 Association of phenolics in MM pepper extracts and bioactivities via WGCNA

**Fig. 5:**
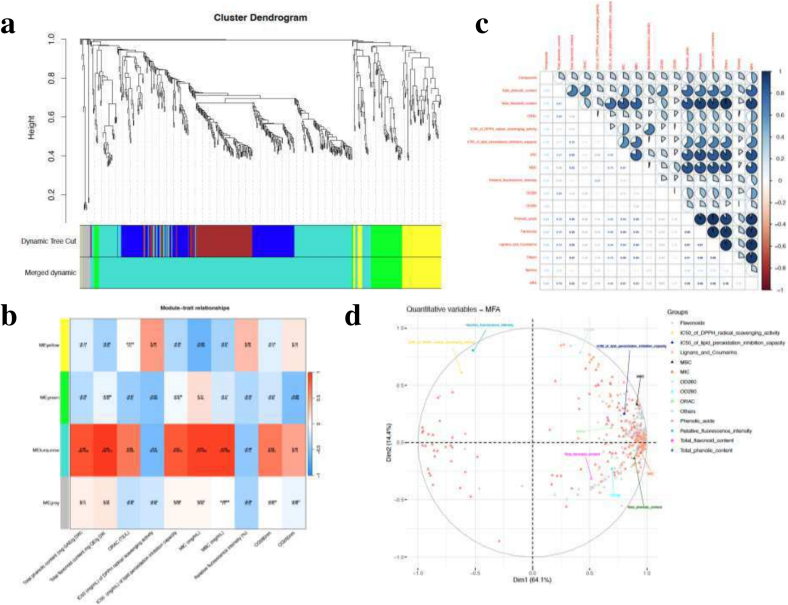
Correlations of phenolics with bioactivities based on WGCNA and MFA. (a) Clustering dendrogram of the average network adjacency for the identification of phenolics co-expression modules. (b) Module-trait relationships. (c) Correlation matrix between variable sets in the MFA analysis. (d) MFA loading plot of antioxidant, antibacterial activities and the phenolics achieved from the key modules.

WGCNA, a correlation-based and unsupervised computational method, was employed to describe and visualize the correlation patterns among data points. To explore the impact of phenolic compounds on the antioxidant and antibacterial activities of pepper extracts, WGCNA was utilized to associate 586 phenolic compounds with antioxidant activity indicators, such as ORAC, IC_50_ of DPPH radical scavenging activity, and IC_50_ values of anti-lipid peroxidation capacity, as well as antibacterial activity indicators including MIC, MBC, relative fluorescence intensity, the absorbance at OD_280_ and OD_260_. After merging, four co-expression modules were identified (Fig. 5a-b). The correlation analysis between the ME of the four modules and the antioxidant and antibacterial activity indicators revealed that the MEturquoise module had the highest correlation with these indicators, encompassing both positive and negative regulatory relationships. It was positively correlated with total phenolic content, total flavonoid content, ORAC, inhibition of lipid oxidation, and leakage of nucleic acids and proteins (*r* > 0.64), and negatively correlated with the IC_50_ of DPPH radical scavenging activity values and relative fluorescence intensity (*r* > 0.46) (Fig. 5b). Among these, the total flavonoid content and antibacterial activity indicators, MIC and MBC, had a positive regulatory correlation as high as above 0.9. In addition, the IC_50_ of DPPH radical scavenging activity and relative fluorescence intensity showed a negative correlation with phenolic compounds.

In addition, MFA was employed to examine the correlation between 436 phenolic compounds in the key turquoise module from WGCNA and antioxidant as well as antimicrobial activities (Fig. 5c). The correlation matrix among variables reveals both the direction and strength of these correlations. Results indicated a significant positive correlation between MIC, MBC, and specific phenolic subclasses, including phenolic acids, flavonoids, lignans, and coumarins (*r* > 0.8). Furthermore, the IC_50_ values for anti-lipid peroxidation, total flavonoid content, ORAC, relative fluorescence content, and OD280 showed correlations with phenolic acids, flavonoids, and tannins (r > 0.6), suggesting potential synergistic effects of these compounds in contributing to antioxidant and antimicrobial activities.

The MFA loading plot reveals that the first two factors (Dim1 and Dim2) account for 78.5 % of the total variation, with Dim1 contributing 64.1 % and Dim2 contributing 14.4 % (Fig. 5d). Most phenolic acids and flavonoids are positioned at opposite ends along the horizontal axis. While the IC_50_ of DPPH radical scavenging activity values and relative fluorescence intensity exhibit a negative correlation, the remaining indicators show a positive correlation. The MFA results highlighted a strong association between the majority of flavonoids and phenolic acids identified in WGCNA and both antioxidant and antibacterial activities. Specifically, MIC, MBC, total flavonoid content, and the IC_50_ values of anti-lipid peroxidation capacity demonstrate high correlations with these compounds. Scatter points concentrated in the central range (−0.5 to 0.5) may represent phenolic compounds with lower Pearson correlation coefficients (∼0.6) in the WGCNA key module. In addition, OD_260_, relative fluorescence intensity, and the IC_50_ of DPPH radical scavenging activity display weaker relationships with the phenolics. These findings align with the module trends observed through WGCNA, further clarifying the roles of key phenolic compounds in the observed activities.

Numerous studies have investigated the phenolic content, antioxidant activity, and antibacterial properties of spices and herbs. For example, previous work demonstrated strong positive linear correlations between antioxidant capacity and total phenolic content in a variety of spices and herbs (Shan et al., 2005). Shan et al. (2007) further examined 46 spice and herb extracts from different regions and found significant linear relationships between antibacterial activity and total phenolic content. This correlation suggests that the antibacterial activity of these extracts was closely related to the concentration of phenolic compounds and their antioxidant capacity. Natural phenolic compounds exhibit antimicrobial activity by disrupting cell membrane integrity, inhibiting the synthesis and metabolism of biomacromolecules, and through other mechanisms (Papuc et al., 2017). Spices and herbs contain diverse phenolic compounds, including phenolic acids, flavonoids, tannins, lignans, coumarins, and quinones. However, the phenolic compounds in many spices and herbs remain insufficiently identified, and the antibacterial mechanisms of individual phenolic compounds are complex. Further research is needed to clarify the relationship between the antibacterial properties of these compounds and their chemical structures within tested extracts.

## 4 Conclusion

Maturity has a significant impact on the composition of substances and the antioxidant activity in berries. Our results showed that as the berries ripened, the TPC, TFC, and antioxidant activity of the free, esterified, glycosylated, and insoluble-bound phenolic extracts of pepper decreased. The phenolic compounds were primarily concentrated in MM stage, which also exhibited the strongest antioxidant capacity. This may be due to changes in the composition and concentration of phenolic and flavonoid compounds during the different stages of ripening.

The four forms of phenolics from MM stage berries were extracted and evaluated as inhibitors to understand their effects on *P. fragi*. The results showed that the insoluble-bound fraction exhibited the strongest bacteriostatic effect, with the lowest MIC and MBC values, followed by the esterified fraction, while the free fraction had the weakest antibacterial effect. Particle size distribution analysis revealed that the treatment of bacterial suspensions with esterified and free fraction led to bacterial aggregation, possibly due to cell membrane damage and adhesion, resulting in larger particle sizes. Further investigation revealed that treatment with the phenolic extracts led to significant leakage of nucleic acids and proteins, along with elevated intracellular ROS levels. The resulting accumulation of toxic free radicals ultimately caused bacterial cell death. Observations from live-dead cell staining and transmission electron microscopy further revealed extensive cell death, with cells showing varying degrees of deformation, membrane rupture, and cytoplasmic leakage.

The results from WGCNA and MFA showed a significant positive correlation between the MIC, MBC, and phenolic acids, flavonoids, lignans, and coumarins. Additionally, the IC_50_ values for anti-lipid peroxidation capacity, total flavonoid content, ORAC, relative fluorescence intensity, and OD_280_ also correlate with phenolic acids, flavonoids, and tannins. This suggests that these compounds may work synergistically to enhance both antioxidant and antibacterial activities.

This was the first systematic study to report a strong positive correlation between antioxidant and antibacterial activity and the phenolic extracts from pepper berries at different maturity stages. The study also demonstrated that pepper extracts were rich in phenolic compounds and exhibited significant antioxidant and antibacterial activities, making them a potential source of inhibitory agents against foodborne pathogens and natural antioxidants. While this study provided valuable insights into the antioxidant and antibacterial properties of pepper phenolic compounds, further research is needed to identify the key antioxidant compounds and major phenolic compounds at various ripening stages.

## CRediT authorship contribution statement

**Yiming Fang:** Writing – original draft, Methodology, Investigation. **Guiping Wu:** Writing – original draft, Methodology, Investigation. **Yunshuang Ding:** Methodology, Investigation, Formal analysis. **Zhiqiang Niu:** Methodology, Investigation, Formal analysis. **Jiale Feng:** Methodology, Investigation. **Rui Guo:** Methodology, Investigation. **Shuzhen He:** Software, Methodology. **Xu Wang:** Methodology, Investigation. **Hongying Zhu:** Methodology, Investigation. **Wenjiang Dong:** Writing – review & editing, Software. **Weicheng Hu:** Writing – review & editing, Methodology. **Xiaojian Jiang:** Methodology, Conceptualization. **Fenglin Gu:** Writing – review & editing, Supervision, Project administration.

## Declaration of competing interest

The authors declare that they have no known competing financial interests or personal relationships that could have appeared to influence the work reported in this paper.

## Data Availability

Data will be made available on request.

## Appendix A Supplementary data

Supplementary material Image 1
